# Predictive value of gut metabolites combined with neutrophil to platelet ratio for clinical functional outcome in acute ischemic stroke treated with endovascular therapy

**DOI:** 10.3389/fnut.2026.1747818

**Published:** 2026-04-30

**Authors:** Rongrong Liu, Wei Li, Chu Zhou, Yukun Wang, Qiyang Yuan, Ruoyu Qin, Rongli Yang, Shiguang Zhu

**Affiliations:** 1 Graduate School of Xuzhou Medical University, Xuzhou, Jiangsu, China; 2 Xinhua Hospital Affiliated to Shanghai Jiao Tong University School of Medicine, Chongming Branch, Shanghai, China; 3 The Affiliated Hospital of Xuzhou Medical University, Xuzhou, Jiangsu, China

**Keywords:** acute ischemic stroke (AIS), endovascular therapy (EVT), gut metabolites, inflammation, novel platelet-derived inflammatory biomarkers, outcome, Trimethylamine N-Oxide (TMAO)

## Abstract

**Purpose:**

This study aimed to investigate the associations between gut metabolites Trimethylamine N-Oxide (TMAO), the novel platelet-derived inflammatory ratio index neutrophil-to-platelet ratio (NPR), and the prognosis of patients with acute ischemic stroke (AIS) undergoing endovascular therapy (EVT).

**Methods:**

This study was a retrospective case–control study. Data were collected from 213 AIS patients who underwent EVT at the Stroke Alliance of the Affiliated Hospital of Xuzhou Medical University between October 2022 and December 2024, including baseline characteristics, laboratory results, and gut-derived metabolite levels from the proximal culprit vessel. Functional outcome was assessed using the modified Rankin scale (mRS) at 3 months after EVT. Based on univariable analysis, a multivariable binary logistic regression model was employed to explore the association of gut-derived TMAO and the platelet-derived inflammatory biomarker NPR with poor functional outcomes. The predictive values of TMAO and NPR, both individually and in combination, were quantitatively compared using receiver operating characteristic (ROC) curves integrated with the DeLong test, continuous net reclassification improvement (cNRI), and integrated discrimination improvement (IDI). Finally, both multiplicative and additive interactions between TMAO and NPR regarding poor functional outcomes were evaluated.

**Results:**

A total of 213 eligible patients were divided into the favorable group (mRS ≤ 3, *N* = 77) and the unfavorable group (mRS > 3, *N* = 136). After adjusting for confounding factors, multivariable logistic regression analysis revealed that age (OR: 1.065, 95% CI: 1.022–1.109, *p* = 0.002), baseline National Institutes of Health Stroke Scale (NIHSS) (OR: 1.069, 95% CI: 1.008–1.133, *p* = 0.025), TMAO (OR: 2.889, 95% CI: 1.563–5.338, *p* < 0.001), NPR (OR: 1.864, 95% CI: 1.122–3.096, *p* = 0.016), onset-to-reperfusion time (OTR) (OR: 1.004, 95% CI: 1.002–1.007, *p* = 0.002), complete recanalization (OR: 0.129, 95% CI: 0.032–0.530, *p* = 0.004), and hemorrhagic transformation(HT) (OR: 3.271, 95% CI: 1.351–7.918, *p* = 0.009) were independent predictors of poor functional outcomes. The areas under the curve (AUC) for TMAO, NPR and the combined value of TMAO and NPR in predicting unfavorable outcomes at 3 months after EVT were 0.698, 0.651 and 0.749, respectively. Furthermore, the DeLong test, along with cNRI and IDI analyses, confirmed the significant incremental predictive value of the combined model, and a significant additive interaction between TMAO and NPR was identified.

**Conclusion:**

This study revealed that elevated levels of TMAO and NPR are independently associated with poor functional outcomes in AIS patients after EVT. The combined assessment of TMAO and NPR provides incremental value in predicting the prognosis of these patients.

## 1 Introduction

Acute ischemic stroke (AIS) primarily arises from cerebrovascular circulatory disturbances, leading to ischemic necrosis of brain tissue and subsequent neurological deficits. Endovascular therapy (EVT) has emerged as a cornerstone therapeutic approach for patients with AIS (1, 2). However, given the substantial heterogeneity in clinical outcomes following EVT, there is an urgent need to identify reliable biomarkers for the early prediction of post-procedural prognosis.

Recent research based on the brain–gut axis theory has demonstrated, through microbial transplantation experiments and clinical studies, that intestinal dysbiosis during the acute phase of stroke can activate platelets and induce inflammatory responses (3). In 2011, a landmark study published in *Nature* identified Trimethylamine N-Oxide (TMAO) as a potent risk factor for cardiovascular diseases (4). Gut microbiota metabolize dietary precursors, such as choline and L-carnitine, into trimethylamine (TMA), which is subsequently oxidized to TMAO in the liver (5). TMAO has been shown to accelerate the progression of atherosclerosis by promoting foam cell formation, disruption of cholesterol and bile acid metabolism, and inducing vascular inflammation and endothelial dysfunction. Furthermore, it significantly increase platelet hyperreactivity and the risk of thrombosis (6–9).

Platelets play a pivotal role in the pathogenesis of AIS and are intricately linked to various pathophysiological processes, including atherosclerosis, thrombosis, and neuroinflammation. Recent clinical studies have demonstrated that novel platelet-derived inflammatory ratio indices hold significant prognostic value in cardiovascular and cerebrovascular conditions, such as coronary heart disease, acute myocardial infarction, and acute stroke (10–13). Consequently, integrating peripheral blood inflammatory cell parameters with platelet counts to derive indices such as the neutrophil-to-platelet ratio (NPR) may offer a novel approach for risk stratification and prognostic evaluation in patients with AIS.

However, to date, no study has thoroughly investigated the associations of gut metabolites and novel platelet-derived inflammatory biomarkers with clinical outcomes in patients treated with EVT. To address this research gap, the present study aimed to evaluate the association of TMAO and NPR levels with short-term outcomes in this population. Additionally, we investigated whether the combination of these two markers exhibits a synergistic interaction in predicting AIS clinical outcomes. This work seeks to provide novel insights into the roles of gut microbiota metabolites and novel inflammatory markers in stroke recovery, potentially guiding risk stratification and the optimization of subsequent therapeutic strategies.

## 2 Materials and methods

This study employed a retrospective observational design utilizing prospectively archived biospecimens. This methodology combined the rigor of prospective sample collection with the efficiency of retrospective data analysis, thereby minimizing potential selection and information biases typically inherent in traditional retrospective studies.

A total of 213 patients with AIS who underwent EVT at the Stroke Alliance of the Affiliated Hospital of Xuzhou Medical University between October 2022 and December 2024 were included in the final analysis.

### 2.1 Inclusion criteria

The inclusion criteria were as follows: (1) patients admitted via the “green channel” for emergency intervention; (2) patients who fulfilled the diagnostic criteria for AIS specified in the *Chinese Guidelines for the Diagnosis and Treatment of Acute Ischemic Stroke 2018*; (3) aged ≥ 18 years; (4) compliance with EVT criteria, with patients or their immediate relatives providing informed consent and undergoing emergency EVT; (5) complete baseline and relevant hematological data; (6) complete clinical follow-up data at 3 months after EVT; and (7) no consumption of medications such as gut microbiota regulators, gastric acid secretion inhibitors or antibiotics within the preceding month.

### 2.2 Exclusion criteria

The exclusion criteria were as follows: (1) modified Thrombolysis in Cerebral Infarction (mTICI) < 2b; (2) severe coagulation dysfunction or pronounced active hemorrhage; (3) complicated with severe impairment of major organs such as the heart, liver, and kidneys; (4) clearly diagnosed hematological disorders, severe infections, malignant tumors, or other conditions that compromise normal bone marrow hematopoiesis; (5) incomplete clinical data; and (6) loss to follow-up or refused to undergo follow-up.

### 2.3 Biospecimen collection and biomarker measurement (prospective phase)

Arterial blood samples were prospectively and consecutively collected during EVT as part of the acute ischemic stroke biobank established by our hospital’s Stroke Alliance Center. The collection followed a strictly standardized protocol: prior to EVT, a microcatheter was advanced to the target lesion under microwire guidance. Subsequently, 5 mL of arterial blood proximal to the thrombus was aspirated using a syringe attached to the microcatheter hub.

Blood was immediately transferred into both clot activator tubes (for serum separation) and EDTA anticoagulant tubes (for plasma). Following centrifugation at 2000 × *g* for 10 min, serum and plasma were separated, aliquoted, and stored at −80 °C until batch analysis. Biomarker quantification was conducted using high-performance liquid chromatography–tandem mass spectrometry (HPLC–MS/MS) to measure plasma concentrations of TMAO, choline, betaine, carnitine, and creatinine. To minimize inter-assay variability and detection bias, all samples were processed in randomized batches by laboratory technicians blinded to the patients’ clinical information and outcomes.

### 2.4 Clinical data extraction (retrospective phase)

Following the formulation of the study hypothesis, clinical data were retrospectively extracted from the electronic medical record (EMR) system and the Stroke Alliance Center’s registry.

Extracted variables included the following: (1) Demographics: age and sex; (2) Vascular risk factors: hypertension, diabetes mellitus, atrial fibrillation, coronary artery disease, previous stroke, current smoking, and alcohol consumption; (3) Clinical and etiologic parameters: admission systolic blood pressure (SBP), diastolic blood pressure (DBP), baseline National Institutes of Health Stroke Scale (NIHSS) score, initial modified Rankin Scale (mRS) score, preprocedural Alberta Stroke Program Early CT Score (ASPECTS), Trial of Org 10,172 in Acute Stroke Treatment (TOAST) classification (including large-artery atherosclerosis [LAA] and cardioembolism [CE]), and occlusion location; (4) Laboratory values: leukocyte count, red blood cell (RBC) count, platelets, neutrophils, lymphocytes, mean platelet volume (MPV), red cell distribution width (RDW), and estimated glomerular filtration rate (eGFR), all derived from venous blood samples collected at admission prior to EVT; and (5) Procedural characteristics: use of bridging therapy, anesthesia strategy, onset-to-reperfusion time (OTR), door-to-reperfusion time (DRT), hemorrhagic transformation(HT), and successful recanalization, defined as a modified Thrombolysis in Cerebral Infarction (mTICI) score of 2b or 3.

### 2.5 Calculation of platelet-derived novel inflammatory indices

The NPR was calculated as the absolute neutrophil count divided by the platelet count. Other platelet-derived indices were defined as follows: the platelet-to-lymphocyte ratio (PLR) was the ratio of platelet counts to lymphocyte count; the mean platelet volume-to-lymphocyte ratio (MPVLR) was the ratio of MPV to lymphocyte count; the white blood cell count-to-MPV ratio (WMR) was the ratio of white blood cell count to MPV; and the platelet-to-white blood cell ratio (PWR) was the ratio of platelet count to white blood cell count.

### 2.6 Follow-up and outcome assessment

The primary outcome was functional status at 3 months. Patients were followed up via telephone or outpatient visits at 3 months post-EVT, and follow-up results were recorded in real-time in the Stroke Alliance Center’s follow-up registry. During this period, all patients received guideline-recommended standardized treatments, including antiplatelet aggregation, lipid regulation, plaque stabilization, and rehabilitation training.

The 3-month mRS scores were independently evaluated by two experienced neurologists. In the event of a disagreement, a third neurologist was consulted to reach a consensus. Based on the ability to walk independently, clinical functional outcomes were dichotomized into a favorable outcome group (mRS scores of 0–3) and an unfavorable outcome group (mRS scores of 4–6).

### 2.7 Ethical approval

The study adhered to the Declaration of Helsinki established by the World Medical Association and received approval from the Institutional Ethics Committee of the Affiliated Hospital of Xuzhou Medical University. (Ref. No. XYFY2025-KL362-01).

### 2.8 Statistical analysis

Statistical analyses were conducted using IBM SPSS Statistics (version 27.0) and R software (version 4.5.2). The Shapiro–Wilk test was employed to assess the normality of continuous variables. Normally distributed data were expressed as means (standard deviation, SD) and compared using the independent samples t-test. Non-normally distributed data were presented as medians (interquartile range, IQR) and analyzed using the nonparametric rank sum test (Mann–Whitney U test). Categorical variables were expressed as frequencies and percentages (*n* [%]) and compared using the chi-square test.

To strictly control the false discovery rate (FDR) in exploratory univariate analyses, *p*-values were adjusted using the Benjamini–Hochberg (BH) method. Considering the extremely small raw values of NPR, which could lead to numerical instability and clinically uninterpretable odds ratios (OR) in logistic models, NPR was linearly rescaled by a factor of 100 prior to inclusion in the regression analysis. Consequently, each 1-unit increase in the transformed variable corresponds to a 0.01 increase in the original NPR value. This linear transformation does not alter the statistical significance or the model’s goodness-of-fit.

Multicollinearity among covariates was assessed using the variance inflation factor (VIF) prior to multivariable regression analysis. To adjust for confounding factors and identify independent predictors, multivariable binary logistic regression was conducted. The predictive performance of TMAO and NPR, both individually and in combination, was quantitatively compared using receiver operating characteristic (ROC) curves integrated with the DeLong test, continuous net reclassification improvement (cNRI), and integrated discrimination improvement (IDI).

To prevent model overfitting and ensure parameter parsimony in the interaction analysis, backward stepwise selection based on the Akaike Information Criterion (AIC) was applied to the interaction models. Subsequently, both multiplicative and additive interactions between TMAO and NPR regarding 3-month functional outcomes were evaluated. Additive interactions were quantified using the relative excess risk due to interaction (RERI), the attributable proportion (AP), and the synergy index (SI). A two-tailed *p* < 0.05 was considered statistically significant.

## 3 Results

### 3.1 Baseline characteristics of the study population

**Figure 1:**
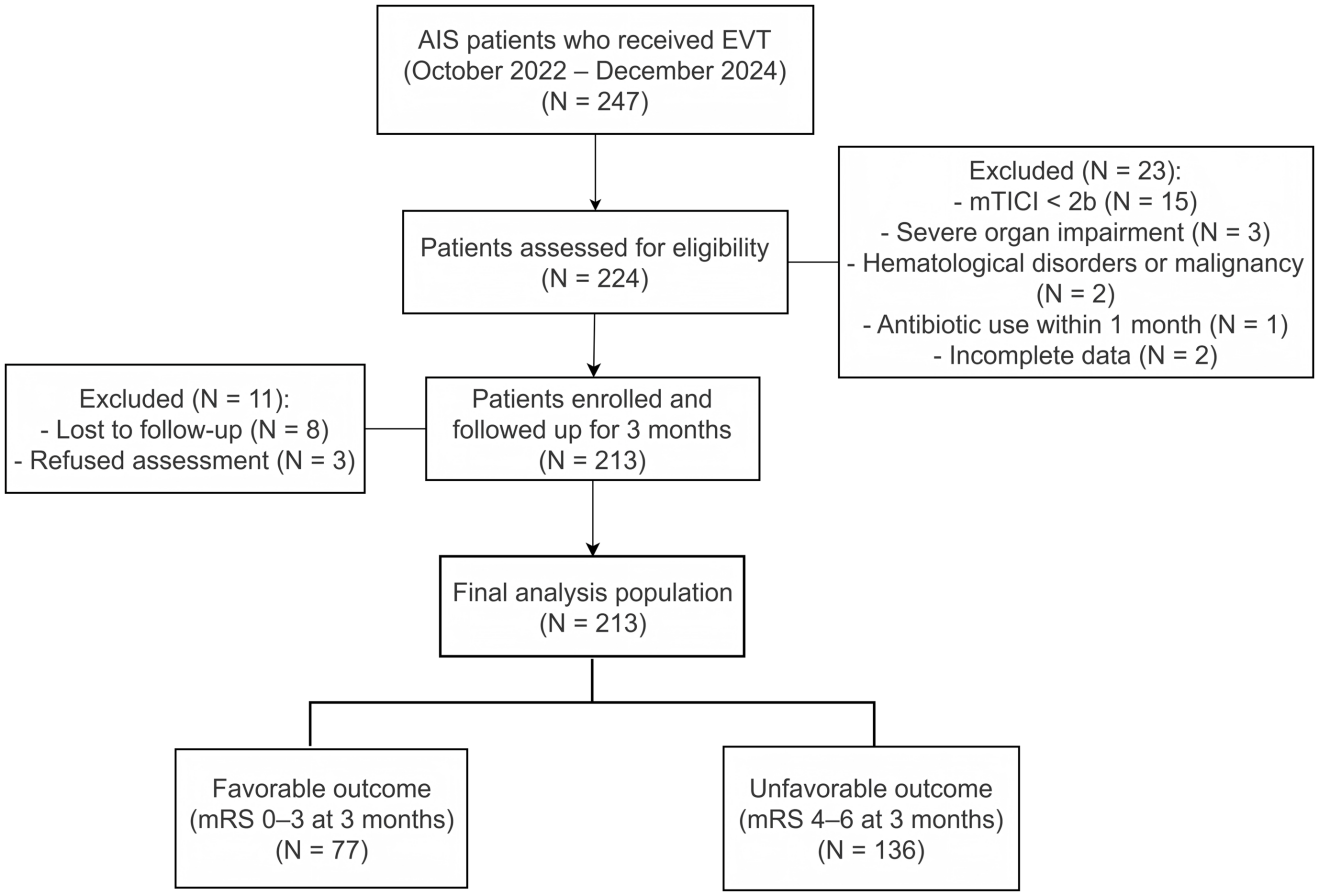
Flowchart of patient screening and enrollment.

Figure 1 illustrates the flowchart of the patient screening and enrollment process. The baseline clinical and procedural characteristics of the study population are summarized in Table 1. Of the 213 enrolled patients, 136 (63.8%) presented with an mRS score of 4–6 and were classified into the unfavorable group while the remaining 77 (36.2%) were categorized into the favorable group.

**Table 1:** Baseline characteristics of the study participants by outcome.

Variable	Favorable group (*n* = 77)	Unfavorable group (*n* = 136)	X^2^/Z/t	*p*	Adjusted *p* value (FDR)
Male, *n*(%)	53 (68.8%)	86 (63.2%)	0.679	0.410	0.545
Age, years, IQR	65 (55.0, 72.0)	70 (63.3, 77.8)	−3.511	<0.001***	0.013*
Hypertension, *n*(%)	42 (54.5%)	79 (58.1%)	0.251	0.616	0.725
Diabetes mellitus, *n*(%)	17 (22.1%)	31 (22.8%)	0.014	0.904	0.904
Atrial fibrillation, *n*(%)	21 (27.3%)	51 (37.5%)	2.298	0.130	0.248
Coronary heart disease, *n*(%)	16 (20.8%)	21 (15.4%)	0.976	0.323	0.497
Previous stroke, *n*(%)	15 (19.5%)	33 (24.3%)	0.645	0.422	0.545
Smoking, *n*(%)	8 (10.4%)	23 (16.9%)	1.682	0.195	0.338
Alcohol use, *n*(%)	6 (7.8%)	21 (15.4%)	2.599	0.107	0.225
SBP, mmHg	148.68 ± 26.73	150.07 ± 24.36	−0.386	0.700	0.778
DBP, mmHg	85 (78.0, 98.0)	85 (77.3, 94.5)	−0.409	0.683	0.778
Baseline NIHSS, score	17 (11.0, 23.5)	20.5 (15.0, 27.0)	−3.066	0.002**	0.013*
Baseline mRS, score	4 (4.0, 4.0)	4 (4.0, 5.0)	−3.122	0.002**	0.013*
DRT, minute	156 (131.5, 214.0)	176 (130.3, 228.3)	−1.216	0.224	0.358
OTR, minute	360 (266.5, 435.0)	410 (320.0, 508.8)	−2.495	0.013*	0.058
Anterior circulation, *n*(%)	56 (72.73%)	100 (73.53%)	0.016	0.899	0.904
Bridging thrombolysis, *n*(%)	41 (53.25%)	70 (51.47%)	0.062	0.803	0.868
LAA, *n*(%)	56 (72.73%)	94 (69.12%)	0.308	0.579	0.702
General anesthesia, *n*(%)	55 (71.43%)	105 (77.21%)	0.878	0.349	0.499
mTICI = 3, *n*(%)	72 (93.51%)	112 (82.35%)	5.200	0.023*	0.083
HT, *n*(%)	15 (19.48%)	52 (38.24%)	8.021	0.005**	0.025*
ASPECT, score	7 (6.0, 8.0)	7 (5.3, 8.0)	−0.175	0.861	0.904

*p* values in the last column were adjusted for multiple comparisons using the Benjamini–Hochberg false discovery rate (FDR) method.

*p < 0.05; **p < 0.01; ***p < 0.001.

SBP, systolic blood pressure; DBP, diastolic blood pressure; baseline NIHSS, baseline National Institutes of Health Stroke Scale (NIHSS) score; baseline mRS, baseline modified Rankin Scale score; DRT, door-to-reperfusion time; OTR, onset-to-reperfusion time; LAA, large-artery atherosclerosis; mTICI, modified Thrombolysis in Cerebral Infarction; HT, hemorrhagic transformation; ASPECTS, Alberta Stroke Program Early CT Score.

Compared with the favorable group, patients in the unfavorable group were significantly older (*p* < 0.001) and exhibited higher baseline NIHSS scores (*p* = 0.002) and initial baseline mRS scores (*p* = 0.002) upon admission. Regarding procedural parameters, the unfavorable group had a significantly longer OTR (*p* = 0.013) and a higher incidence of HT (*p* = 0.005). In contrast, a significantly higher proportion of complete recanalization (mTICI = 3) was observed in the favorable outcome group (*p* = 0.023). Notably, after FDR correction, age, baseline NIHSS score, baseline mRS score, and HT remained significantly different between the two groups (all adjusted *p* < 0.05). No signifcant diference were observed between the two groups regarding sexes, smoking, alcohol consumption, vascular risk factors (hypertension, diabetes mellitus, atrial fibrillation, coronary heart disease, and previous stroke), or admission blood pressure (all *p* > 0.05). Furthermore, no significant disparities were found in terms of occlusion location (anterior vs. posterior circulation), anesthesia strategy, use of bridging therapy, TOAST classification (LAA vs. CE), preprocedural ASPECTS, or DRT (all *p* > 0.05).

### 3.2 Comparison of laboratory parameters between the two groups

Regarding the laboratory parameters, the initial unadjusted analysis indicated that patients in the unfavorable group had significantly lower levels of RBC (*p* = 0.025) and platelets (*p* = 0.032) compared to those in the favorable group. However, these differences did not reach statistical significance after the FDR correction (Adjusted *p* value > 0.05). No statistically significant differences were observed in other baseline laboratory markers (all *p* > 0.05) (Table 2).

**Table 2:** Comparison of laboratory parameters between the two groups.

Variable	Favorable group (*n* = 77)	Unfavorable group (*n* = 136)	Z/t	*p*	Adjusted *p* value (FDR)
WBC (×10^9^/L)	8.40 (6.50, 11.10)	9.1 (7.00, 11.10)	−1.274	0.203	0.338
RBC (×10^12^/L)	4.46 ± 0.55	4.27 ± 0.63	2.254	0.025*	0.083
N (×10^9^/L)	6.7 (4.85, 9.02)	7.30 (5.56, 9.60)	−1.941	0.052	0.149
L (×10^9^/L)	1.1 (0.80, 1.70)	1 (0.7, 1.38)	−1.815	0.070	0.187
PLT (×10^9^/L)	202.51 ± 64.05	183.99 ± 58.10	2.153	0.032*	0.098
MPV (fL)	10.16 ± 1.22	10.33 ± 1.30	−0.957	0.340	0.499
RDW (%)	12.8 (12.3, 13.4)	13 (12.6, 13.5)	−1.517	0.129	0.248
eGFR [ml/(min·1.73m^2^)]	120.00 (97.28,120.00)	118.65 (86.56,120.00)	−1.305	0.192	0.338

eGFR values exceeding 120 mL/(min·1.73 m^2^) were recorded as 120 ml/(min·1.73 m^2^) according to the laboratory reporting limit of our institution.

*p* values in the last column were adjusted for multiple comparisons using the Benjamini–Hochberg false discovery rate (FDR) method.

*p < 0.05.

WBC, white blood cell; RBC, red blood cell; N, neutrophil; L, lymphocyte; PLT, platelet; MPV, mean platelet volume; RDW, red blood cell distribution width; eGFR, estimated glomerular filtration rate.

### 3.3 Comparison of gut microbiota-related metabolites between groups

**Figure 2:**
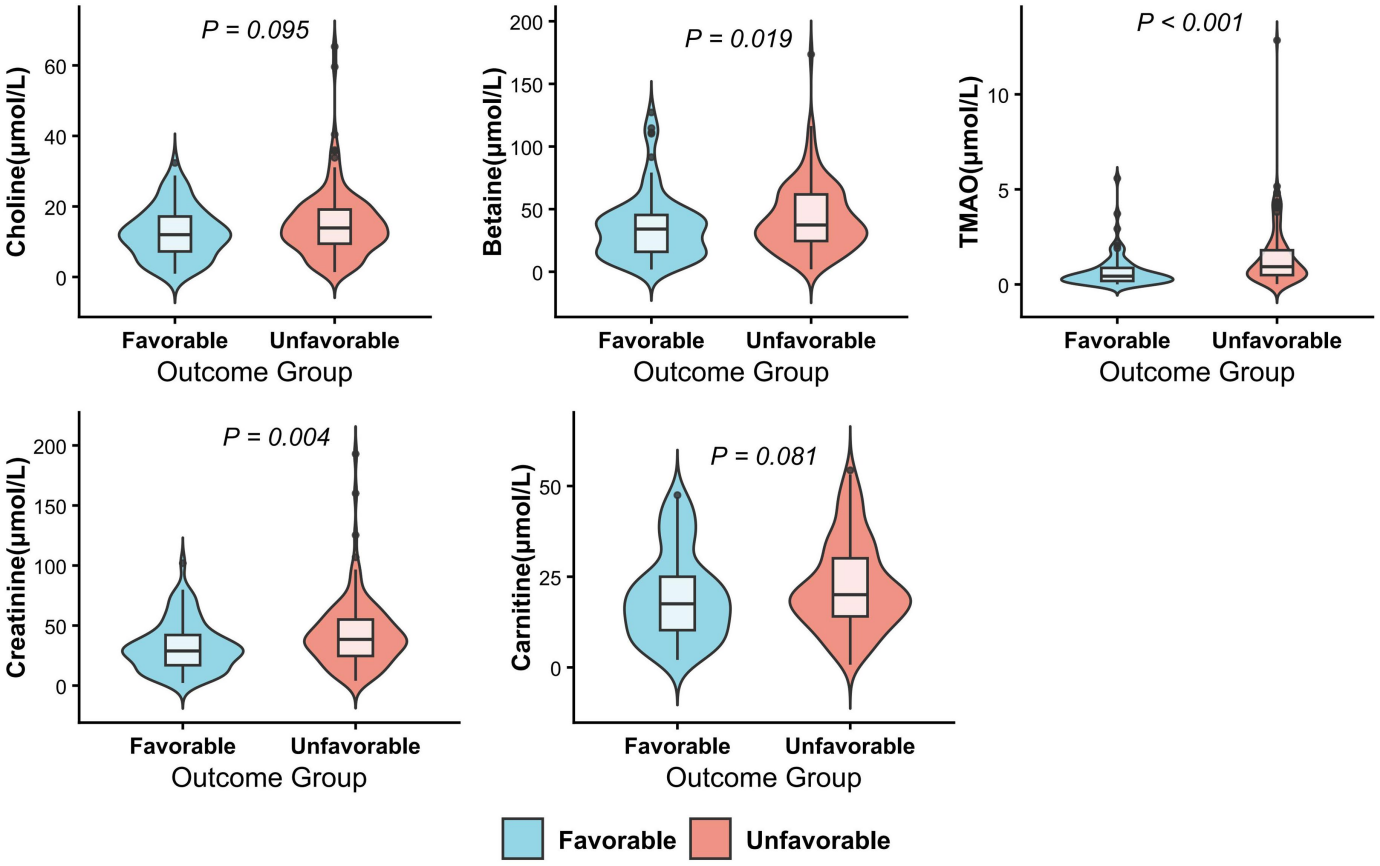
Distribution of gut microbiota-related metabolites between the favorable and unfavorable outcome groups.

Regarding the gut microbiota metabolites measured in arterial blood, the unadjusted analysis revealed significant differences in levels of betaine (*p* = 0.019), TMAO (*p* < 0.0001) and creatinine (*p* = 0.004) between the two groups. Notably, after applying the FDR correction, TMAO remained significantly different between the favorable and unfavorable outcome groups (Adjusted p = 0.004). Creatinine also maintained its statistical significance (Adjusted *p* = 0.023), whereas the difference in betaine levels was no longer significant after correction (Adjusted *p* = 0.076). No significant disparities were found for choline or carnitine (all *p* > 0.05) (Table 3; Figure 2).

**Table 3:** Comparison of gut metabolites between the two groups.

Variable	Favorable group (*n* = 77)	Unfavorable group (*n* = 136)	Z	*p*	Adjusted *p* value (FDR)
Choline, μmol/L	11.99 (7.21, 17.31)	13.90 (9.38, 19.49)	−1.668	0.095	0.211
Betaine, μmol/L	34.10 (15.23, 45.46)	37.33 (24.24, 62.20)	−2.344	0.019*	0.076
TMAO, μmol/L	0.43 (0.18, 0.87)	0.93 (0.49, 1.83)	−4.788	<0.0001****	0.004**
Creatinine, μmol/L	28.83 (16.22, 43.26)	38.47 (23.61, 55.44)	−2.897	0.004**	0.023*
Carnitine, μmol/L	17.53 (10.16, 25.08)	20.04 (14.03, 30.10)	−1.747	0.081	0.203

*p* values in the last column were adjusted for multiple comparisons using the Benjamini–Hochberg false discovery rate (FDR) method.

*p < 0.05; **p < 0.01; ****p < 0.0001.

TMAO, Trimethylamine N-Oxide.

### 3.4 Comparison of platelet-derived ratios between the two groups

**Figure 3:**
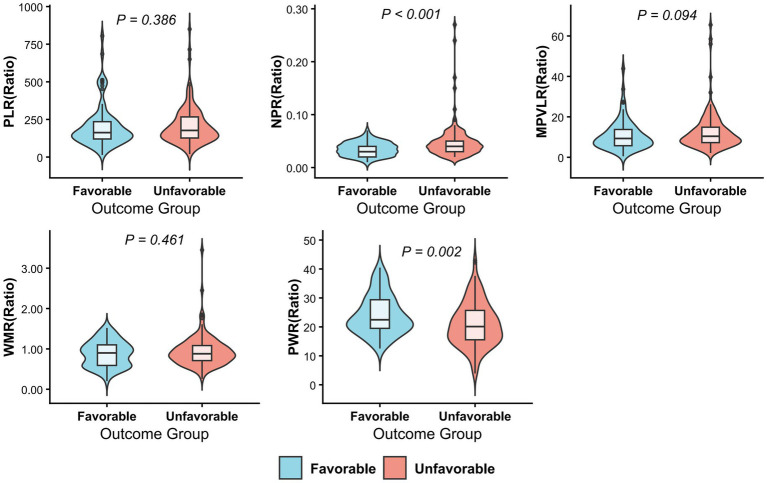
Violin plots comparing novel platelet-derived inflammatory biomarkers between groups.

The analysis of novel platelet-derived inflammatory biomarkers revealed significant differences in several ratios between the two groups. Specifically, patients in the unfavorable group exhibited a significantly higher NPR compared to those in the favorable group (*p* < 0.001). Conversely, the PWR was significantly lower in the unfavorable group (*p* = 0.002). Notably, both NPR and PWR maintained their statistical significance after FDR correction (both adjusted *p* = 0.013). No statistically significant differences were observed in PLR, MPVLR, or WMR (all *p* > 0.05) (Table 4). Violin plots were constructed to visualize the distribution of PLR, NPR, MPVLR, WMR, and PWR between the favorable and unfavorable groups (Figure 3).

**Table 4:** Comparison of novel platelet-derived inflammatory biomarkers between the two groups.

Variable	Favorable group (*n* = 77)	Unfavorable group (*n* = 136)	Z	*p*	Adjusted *p* value (FDR)
PLR, median (IQR)	162.50 (118.95, 236.50)	176.89 (126.52, 267.02)	−0.868	0.386	0.532
NPR, median (IQR)	0.03 (0.02, 0.04)	0.04 (0.03, 0.05)	−3.661	<0.001***	0.013*
MPVLR, median (IQR)	9.3 (5.59, 13.88)	10.39 (7.15, 14.96)	−1.677	0.094	0.211
WMR, median (IQR)	0.90 (0.59, 1.11)	0.88 (0.71, 1.09)	−0.737	0.461	0.576
PWR, median (IQR)	22.46 (19.38, 29.42)	20.11 (15.45, 25.69)	−3.147	0.002**	0.013*

*p* values in the last column were adjusted for multiple comparisons using the Benjamini–Hochberg false discovery rate (FDR) method.

**p < 0.01; ***p < 0.001.

PLR, platelet -to-lymphocyte ratio; NPR, neutrophil-to-platelet ratio; MPVLR, MPV-to-lymphocyte ratio; WMR, white blood cell -to- MPV ratio; PWR, platelet -to- white blood cell ratio.

### 3.5 Multivariate logistic regression analysis for functional outcomes

Prior to the regression analysis, multicollinearity diagnostics were performed. All variables demonstrated VIF values< 5, indicating the absence of significant multicollinearity (Table 5). Variables showing statistical significance in the univariate analysis were subsequently incorporated into a binary multivariate logistic regression model. The model was adjusted for known potential confounders, including sex, eGFR, vascular risk factors, TOAST classification, bridging therapy, anesthesia strategy, occlusion location, OTR, ASPECTS, mTICI, and HT.

**Table 5:** Multicollinearity diagnostics of variables included in the multivariate model.

Variable	Tolerance	VIF
Age	0.612	1.634
Sex	0.779	1.283
Baseline mRS	0.641	1.560
Baseline NIHSS	0.615	1.626
Hypertension	0.806	1.241
Diabetes mellitus	0.867	1.154
Atrial fibrillation	0.560	1.787
Coronary artery disease	0.879	1.138
Previous stroke	0.856	1.168
Smoking	0.752	1.330
Alcohol consumption	0.798	1.253
TOAST	0.569	1.757
Bridging therapy	0.894	1.118
eGFR	0.734	1.363
OTR	0.821	1.218
ASPECT	0.795	1.257
Anesthesia strategy	0.815	1.227
Occlusion location	0.663	1.508
mTICI	0.932	1.073
Betaine	0.395	2.533
TMAO	0.494	2.024
Creatinine	0.333	3.000
NPR	0.584	1.712
PWR	0.572	1.749
HT	0.895	1.117

baseline mRS, baseline modified Rankin Scale score; baseline NIHSS, baseline National Institutes of Health Stroke Scale (NIHSS) score; TOAST, Trial of Org 10,172 in Acute Stroke Treatment; eGFR, estimated glomerular filtration rate; OTR, onset-to-reperfusion time; ASPECTS, Alberta Stroke Program Early CT Score; mTICI, modified Thrombolysis in Cerebral Infarction; TMAO, Trimethylamine N-Oxide; NPR, neutrophil-to-platelet ratio; PWR, platelet-to-white blood cell ratio; HT, hemorrhagic transformation; VIF, variance inflation factor.

**Figure 4:**
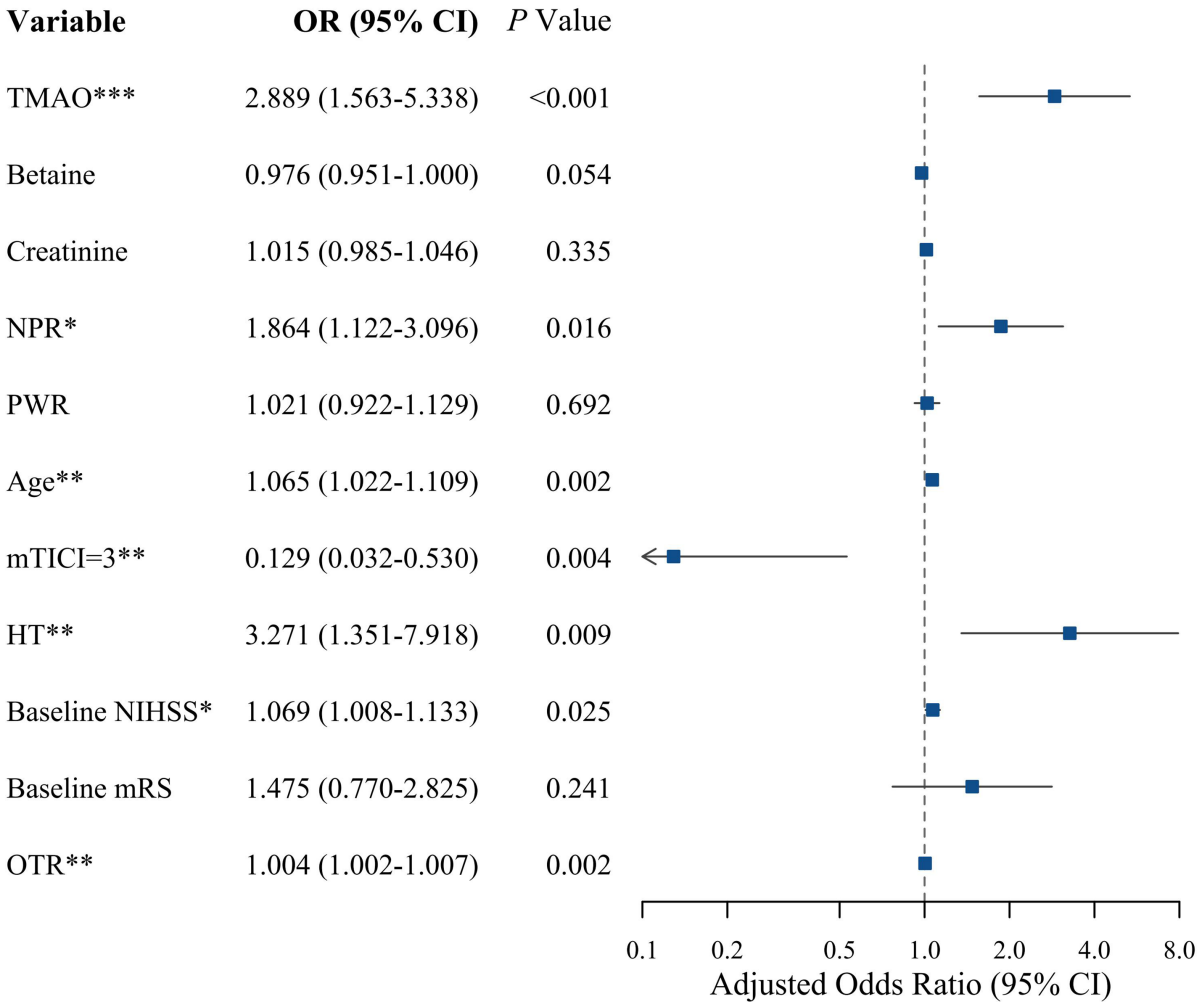
Forest plot of independent predictors for unfavorable prognosis.

After adjusting for these confounding factors, the multivariable analysis revealed that age (OR: 1.065, 95% CI: 1.022–1.109, *p* = 0.002), baseline NIHSS (OR: 1.069, 95% CI: 1.008–1.133, *p* = 0.025), TMAO (OR: 2.889, 95% CI: 1.563–5.338, *p* < 0.001), NPR (OR: 1.864, 95% CI: 1.122–3.096, *p* = 0.016), OTR (OR: 1.004, 95% CI: 1.002–1.007, p = 0.002), and HT (OR: 3.271, 95% CI: 1.351–7.918, *p* = 0.009) were independent risk predictors of unfavorable functional outcomes. Specifically, for every 0.01-unit increment in NPR, the odds of an unfavorable prognosis increased by 86.4%. Conversely, complete recanalization (mTICI = 3) (OR: 0.129, 95% CI: 0.032–0.530, *p* = 0.004) emerged as an independent protective factor against unfavorable outcomes (Table 6). The independent predictors and their corresponding odds ratios (ORs) for unfavorable functional outcomes are further visualized in the forest plot (Figure 4).

**Table 6:** Multivariate logistic regression analysis for predictors of unfavorable functional outcomes.

Variable	*β*	$S_{\bar{x}}$	Wald x^2^	OR (95% CI)	P
Age	0.063	0.021	9.182	1.065 (1.022–1.109)	0.002**
Baseline NIHSS	0.066	0.030	5.005	1.069 (1.008–1.133)	0.025*
Baseline mRS	0.389	0.332	1.375	1.475 (0.770–2.825)	0.241
NPR	0.622	0.259	5.776	1.864 (1.122–3.096)	0.016*
PWR	0.020	0.052	0.157	1.021 (0.922–1.129)	0.692
Betaine	−0.025	0.013	3.709	0.976 (0.951–1.000)	0.054
TMAO	1.061	0.313	11.469	2.889 (1.563–5.338)	<0.001***
Creatinine	0.015	0.015	0.930	1.015 (0.985–1.046)	0.335
OTR	0.004	0.001	9.676	1.004 (1.002–1.007)	0.002**
mTICI = 3	−2.046	0.720	8.080	0.129 (0.032–0.530)	0.004**
HT	1.185	0.451	6.902	3.271 (1.351–7.918)	0.009**

*p < 0.05; **p < 0.01; ***p < 0.001.

OR, odds ratio; CI, confidence interval; baseline NIHSS, baseline National Institutes of Health Stroke Scale (NIHSS) score; baseline mRS, baseline modified Rankin Scale score; NPR,neutrophil-to-platelet ratio; PWR, platelet-to-white blood cell ratio; TMAO, Trimethylamine N-Oxide; OTR, onset-to-reperfusion time; mTICI, modified Thrombolysis in Cerebral Infarction; HT, hemorrhagic transformation.

### 3.6 Predictive performance and incremental value of TMAO and NPR

**Figure 5:**
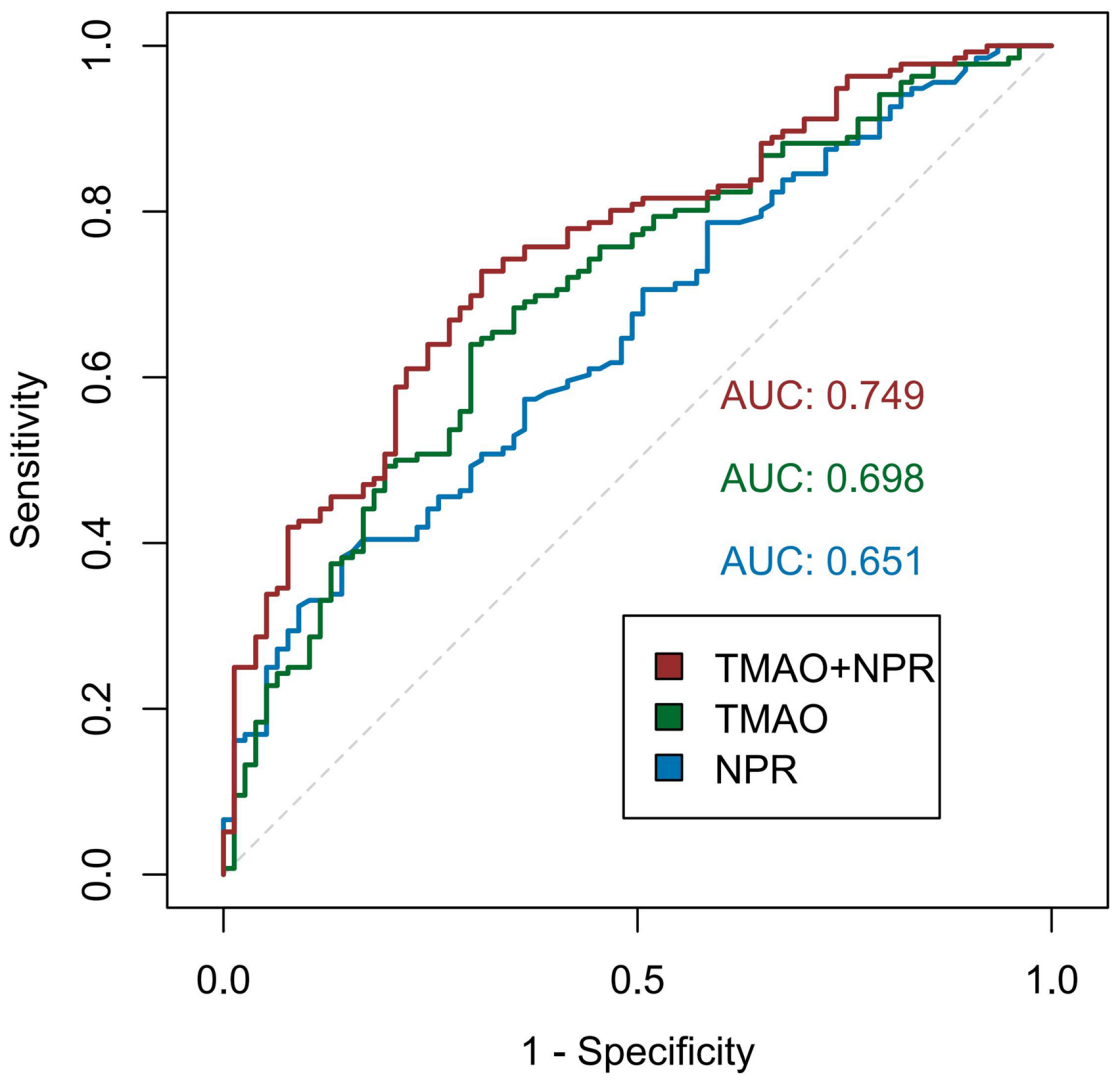
ROC curves of NPR, TMAO, and their combined index for predicting unfavorable outcomes.

ROC curves analysis was performed to evaluate the discriminative capacity of TMAO, NPR, and their combined index in predicting unfavorable outcomes after EVT (Figure 5). The area under the ROC curve (AUC) of TMAO was 0.698 (95% CI: 0.625–0.770), with an optimal cut-off value of 0.683 (sensitivity: 64.0%; specificity: 70.1%). For NPR, the AUC was 0.651 (95% CI: 0.577–0.726), with a cut-off value of 4.675 (sensitivity of 40.4%, specificity of 84.4%). Notably, the combined index (TMAO + NPR) demonstrated superior predictive performance, yielding an AUC of 0.749 (95% CI: 0.683–0.816) (see Table 7).

**Table 7:** Predictive performance of TMAO, NPR, and the combined index for unfavorable outcomes.

Variable	AUC	95% CI	Sensitivity	Specificity	Youden	*p*
NPR	0.651	0.576–0.726	0.382	0.857	0.239	<0.001***
TMAO	0.698	0.625–0.770	0.640	0.701	0.341	<0.001***
TMAO+NPR	0.749	0.683–0.816	0.728	0.688	0.416	<0.001***

***p < 0.001.

AUC, area under the ROC curve; CI, confidence interval; NPR, neutrophil-to-platelet ratio; TMAO, Trimethylamine N-Oxide.

Comparative analysis using the DeLong test showed that the combined index significantly outperformed the single NPR index (AUC improvement: 0.098, 95% CI: 0.035–0.162; *p* = 0.002). Although the combined index exhibited a numerical increase of 0.052 in AUC compared to TMAO alone, the difference did not reach statistical significance via the DeLong test (*p* = 0.113). Since the DeLong test often lacks sensitivity in detecting incremental improvements in AUC when comparing nested models, we further calculated the cNRI and IDI. The addition of NPR to the TMAO-based model resulted in a cNRI of 0.420 (*p* = 0.002) and an IDI of 0.086 (*p* < 0.001), indicating a substantial and clinically meaningful improvement in risk reclassification (Table 8).

**Table 8:** Incremental predictive value of NPR and TMAO for risk reclassification and discrimination.

Comparison	ΔAUC (95% CI)	P_DeLong_	cNRI (95% CI)	P_NRI_	IDI (95% CI)	P_IDI_
Combined index vs. NPR	0.098 (0.035–0.162)	0.002**	0.684 (0.440–0.928)	<0.001***	0.097 (0.060–0.135)	<0.001***
Combined index vs. TMAO	0.052 (−0.012–0.116)	0.113	0.420 (0.148–0.692)	0.002**	0.086 (0.049–0.123)	<0.001***
TMAO vs. NPR	0.046 (−0.066–0.158)	0.416	0.109 (−0.170–0.388)	0.443	0.011 (−0.039–0.062)	0.660

**p < 0.01; ***p < 0.001.

AUC, area under the ROC curve; CI, confidence interval; NPR, neutrophil-to-platelet ratio; TMAO, Trimethylamine N-Oxide; cNRI, continuous net reclassification improvement; IDI, integrated discrimination improvement.

### 3.7 Multiplicative and additive interaction between TMAO and NPR

Based on their optimal cut-off values, TMAO and NPR were categorized into high and low groups, respectively. To prevent model overfitting, backward stepwise selection based on the AIC was applied to screen covariates for the adjusted interaction model. This iterative approach eliminates variables with minimal contributions until the minimum AIC is achieved, ensuring parameter parsimony while maintaining model goodness-of-fit. Ultimately, 10 variables were included in the final interaction model, including TMAO, NPR, age, baseline NIHSS score, coronary artery disease, alcohol consumption, TOAST classification, OTR, mTICI, and HT.

Although no significant multiplicative interaction was identified between TMAO and NPR (*p* = 0.417), we further explored their interaction on an additive scale. The subgroup with both high TMAO and high NPR exhibited a markedly elevated risk of unfavorable outcomes (OR: 78.00; 95% CI: 8.56–711.11) (Table 9). The wide confidence interval observed in this subgroup may be attributed to the limited sample size within this specific category.

**Table 9:** Odds ratios for unfavorable functional outcomes across subgroups stratified by TMAO and NPR levels.

Variable	Case, *n*(%)	OR (95% CI)	*p*
Low TMAO and Low NPR (Reference)	66 (30.9%)	1 (Reference)	NA
Low TMAO and High NPR	37 (17.4%)	5.23 (1.90–14.40)	0.001
High TMAO and Low NPR	84 (39.4%)	5.51 (2.34–12.96)	<0.001***
High TMAO and High NPR	26 (12.2%)	78.00 (8.56–711.11)	<0.001***

***p < 0.001.

OR, odds ratio; CI, confidence interval; NPR, neutrophil-to-platelet ratio; TMAO, Trimethylamine N-Oxide.

Regarding the additive interaction indicators, although the SI showed a significant positive trend (*p* = 0.028) despite a wide confidence interval that marginally crossed 1 (95% CI 0.95–81.42), the AP provided the most robust evidence of interaction. The AP was highly significant (AP: 0.88, 95% CI 0.6–1.15, *p* < 0.001). These findings suggest a robust synergistic effect, where 88% of the risk for unfavorable outcomes in the subgroup with concurrently elevated TMAO and NPR can be attributed to the interaction between these two biomarkers (Table 10).

**Table 10:** Interaction analysis between TMAO and NPR on multiplicative and additive scales.

Measures	Estimate	95% CI	*p*
Multiplicative Scale	2.71	0.24–30.01	0.417
Relative Excess Risk Due to Interaction(RERI)	68.26	−102.33 to 238.86	0.216
Attributable Proportion (AP)	0.88	0.60–1.15	<0.001***
Synergy Index (SI)	8.81	0.95–81.42	0.028*

*p < 0.05; ***p < 0.001.

CI, confidence interval; NPR, neutrophil-to-platelet ratio; TMAO, Trimethylamine N-Oxide.

## 4 Discussion

To the best of our knowledge, this study is the first to investigate the associations between gut metabolites, novel platelet-derived inflammatory biomarkers, and short-term clinical outcomes in patients with AIS following EVT. A unique aspect of our study was the collection of proximal arterial blood directly from the affected vessel during the procedure. This approach allowed us to more accurately assess the concentrations of these metabolites near the thrombus site and their potential role in stroke pathophysiology. Our findings demonstrate that both elevated arterial TMAO and elevated venous NPR are independently associated with unfavorable functional outcomes at 3-month post-EVT. The underlying mechanisms linking these biomarkers to poor prognosis are likely multifaceted and may involve the following complex interactions.

Preclinical studies using animal models have demonstrated that gut microbiota dysbiosis following AIS increases intestinal permeability and triggers systemic immune response. Consequently, gut-derived bacteria and pro-inflammatory mediators translocate across the compromised blood–brain barrier into the brain parenchyma, thereby exacerbating ischemic injury (14). Conversely, specific microbiota-derived metabolites, such as short-chain fatty acids, exert neuroprotective effects by mitigating the post-stroke inflammatory cascade (15).

Dietary precursors rich in choline and L-carnitine are metabolized by gut microbiota and ultimately oxidized into TMAO. Extensive evidence indicates that TMAO accelerates atherosclerosis via multiple pathways, including the promotion of foam cell formation, disruption of cholesterol and bile acid metabolism, as well as the induction of vascular inflammation and endothelial dysfunction (16–18). Furthermore, TMAO enhances platelet hyperreactivity and heightens the risk of thrombosis, making its systemic fluctuations a critical driver in the pathogenesis and progression of cardiovascular and cerebrovascular disease (19).

Previous case–control studies have indicated that elevated TMAO levels are correlated with a heightened risk of stroke in community populations (20). Consistent with these findings, our retrospective analysis revealed that patients with AIS in the unfavorable outcome group exhibited elevated TMAO levels compared with those in the favorable outcome group. This suggests that TMAO levels are essential for assessing the short-term clinical outcomes of patients following EVT.

Numerous previous studies have confirmed that inflammation plays a pivotal role in the onset and progression of AIS (21, 22). Neutrophils can generate free radicals, induce brain damage, and participate in thrombosis, thereby impairing post-stroke revascularization and vascular remodeling, which exacerbates neuronal ischemic injury (23, 24). Consequently, elevated neutrophil in the acute phase are indicative of a poor prognosis (25). Furthermore, damage to the vascular endothelium activates platelets, which release inflammatory mediators that trigger a cascade of reactions, including platelet aggregation and vasospasm, thereby accelerating atherosclerosis (26, 27). Turbulent blood flow near arterial plaques further aggravates platelet activation, creating a pathological “inflammation–thrombosis” cycle that ultimately results in acute cerebrovascular occlusion.

NPR can be derived from routine blood tests that are readily accessible in clinical settings. As a composite marker, NPR reflects the balance between platelets and neutrophils, bridging the mechanisms of thrombosis and inflammation. Previous studies have confirmed that NPR functions as an independent predictor for a 90-day unfavorable outcomes in patients with AIS (28–30), while the interaction between platelets and neutrophils exacerbates the inflammatory response during thrombus formation (31). Patients with AIS may experience a reduction in peripheral blood platelet count due to continuous platelet activation and subsequent depletion. Such a decline in circulating platelets may signify extensive infarction, HT, or the emergence of other detrimental events (32). Consequently, the consumption of platelets during thrombosis, coupled with the elevation of neutrophils resulting from systemic inflammation, contribute to a rise in NPR.

Furthermore, NPR is associated with an increased risk of fatal stroke in middle-aged and elderly patients (29, 33). It acts as an independent predictor of HT in patients following intravenous thrombolysis (34). HT was significantly associated with a 3.271-fold higher risk of unfavorable outcomes (OR: 3.271, 95% CI: 1.351–7.918, *p* = 0.009), reinforcing the link between post-procedural complications and poor prognosis. Our results demonstrated that after adjusting for known confounding factors, NPR remained independently correlated with a short-term adverse prognosis (OR: 1.864, 95% CI: 1.122–3.096, *p* = 0.016), which is consistent with findings from previous work. This suggests that NPR may reflect the underlying inflammatory-thrombotic burden that predisposes patients to HT, thereby contributing to compromised functional recovery following EVT.

Although both TMAO and NPR have been established as independent risk factors for poor prognosis in patients with AIS, no prior study has investigated the combined predictive value of these biomarkers in the context of EVT. Therefore, our study conducted a comprehensive analysis of the gut-derived metabolite TMAO and the novel platelet-derived inflammatory index NPR. The AUC for the combined index was significantly larger than that of either biomarker alone. Furthermore, the DeLong test, along with cNRI and IDI analyses, confirmed the significant incremental predictive value of the combined index. Beyond these improvements in discrimination and reclassification, a significant additive interaction between TMAO and NPR was identified. Our results revealed a robust synergistic effect between TMAO and NPR in predicting outcomes for patients with AIS following EVT (AP: 0.88, 95%CI 0.6–1.15, *p* < 0.001).

Additionally, Multivariate binary logistic regression analysis showed that advanced age was correlated with poor stroke prognosis, with each one-year increase in age associated with a 1.065-fold increased risk of unfavorable outcomes (OR: 1.065, 95% CI: 1.022–1.109, *p* = 0.002). This finding is consistent with established literature identifying age as a critical determinant of functional recovery. Extended OTR potentially amplifies the synergistic impact of TMAO and NPR on neural injury due to prolonged ischemia, whereas complete revascularization remains a key determinant of favorable outcomes.

This study was conducted under the auspices of the Stroke Specialty Alliance of the Affiliated Hospital of Xuzhou Medical University, ensuring a standardized approach to patient treatment and management. However, several limitations should be acknowledged. First, as a retrospective study centered in Jiangsu Province, the geographical scope and sample size may limit the generalizability of our findings to broader populations. Second, our analysis focused exclusively on patients who achieved successful recanalization, which might restrict the applicability of our conclusions to the entire spectrum of patients undergoing EVT. Third, TMAO and NPR levels were measured at a single time point without dynamic monitoring; furthermore, TMAO analysis was confined to arterial blood from the affected vessel, and a direct comparison with systemic venous blood has not yet been performed. Fourth, although we adjusted for major confounders, certain variables such as dietary habits, pre-existing infections, and specific EVT procedural nuances were not standardized. Fifth, due to the inherent constraints of retrospective data, advanced imaging parameters such as ischemic penumbra and core infarct volume were not incorporated into our predictive models. Finally, this study lacks external validation. Therefore, large-scale, multicenter prospective cohorts are warranted to further verify the synergistic predictive value of TMAO and NPR in stroke prognosis.

## 5 Conclusion

In conclusion, the combined prediction of TMAO and NPR can effectively evaluate the 3-month prognosis of patients with AIS after EVT, demonstrating superior predictive value compared with either biomarker alone. The combined prediction of the two comprehensively accounts for the effects of three types of indicators, namely, gut metabolites, platelets, and inflammatory indicators, on the prognosis of patients with AIS. This facilitates risk classification of patients, and guides personalized treatment.

## Data Availability

The raw data supporting the conclusions of this article will be made available by the authors, without undue reservation.
